# Evaluating the prevalence and severity of metabolic dysfunction‐associated steatotic liver disease in patients with type 2 diabetes mellitus in primary care

**DOI:** 10.1111/joim.20103

**Published:** 2025-06-16

**Authors:** Wile Balkhed, Martin Bergram, Fredrik Iredahl, Markus Holmberg, Carl Edin, Carl‐Johan Carlhäll, Tino Ebbers, Pontus Henriksson, Christian Simonsson, Karin Rådholm, Gunnar Cedersund, Mikael Forsgren, Olof Dahlqvist Leinhard, Cecilia Jönsson, Peter Lundberg, Stergios Kechagias, Nils Dahlström, Patrik Nasr, Mattias Ekstedt

**Affiliations:** ^1^ Department of Health Medicine and Caring Sciences Linköping University Linköping Sweden; ^2^ Primary Health Care Center Department of Health Medicine and Caring Sciences Linköping University Linköping Sweden; ^3^ Faculty of Medicine and Health Sciences Linköping University Linköping Sweden; ^4^ Wallenberg Center for Molecular Medicine (WCMM) Linköping University Linköping Sweden; ^5^ Center for Medical Image Science and Visualization (CMIV) Linköping University Linköping Sweden; ^6^ AMRA Medical AB Linköping Sweden; ^7^ Department of Radiation Physics and Department of Health Medicine and Caring Sciences Linköping University Linköping Sweden; ^8^ Department of Radiology and Department of Health Medicine and Caring Sciences Linköping University Linköping Sweden

**Keywords:** fibrosis, MASLD, myosteatosis, obesity, primary care, sarcopenia, Type 2 diabetes mellitus

## Abstract

**Background and aims:**

The prevalence of metabolic dysfunction‐associated steatotic liver disease (MASLD) has increased during the epidemic of obesity. Type 2 diabetes mellitus (T2DM) is associated with progressive MASLD. Therefore, many guidelines recommend screening for MASLD in patients with T2DM. Most studies in patients with MASLD have been conducted in specialist care. We investigated the prevalence and severity of MASLD in patients with T2DM from primary care.

**Methods:**

Patients with T2DM were prospectively included from primary care facilities to undergo transient elastography with controlled attenuation parameter and whole‐body magnetic resonance imaging (MRI) to assess liver fat, cardiac function, muscle composition, and distribution of body fat.

**Results:**

Among 308 participants, 59% had MASLD, 7% had suspected advanced fibrosis (transient elastography ≥ 10 kPa), and 1.9% had cirrhosis. The mean age was 63.9 ± 8.1 years; 37% were female, with no differences between the MASLD and the non‐MASLD groups. Participants with MASLD had greater body mass index (31.1 ± 4.4 vs. 27.4 ± 4.1 kg/m^2^, *p *< 0.001) and a higher prevalence of obesity (60% vs. 21%, *p* < 0.001). Obesity increased the risk of fibrotic MASLD eightfold, as confirmed by multivariable analysis. Participants with MASLD also had increased visceral and abdominal subcutaneous adipose tissue and muscle fat infiltration. On cardiac MRI, participants with MASLD had a lower left ventricular (LV) stroke volume index, a lower LV end‐diastolic volume index, and an increased LV concentricity.

**Conclusions:**

In this cohort of primary care patients with T2DM, 59% had MASLD, and 7% had suspected advanced fibrosis. Obesity was a strong predictor of fibrotic MASLD. MASLD was associated with alterations to the left ventricle and increased deposition of ectopic fat.

## Graphical abstract

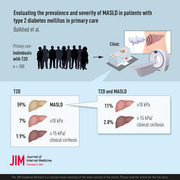

**Abbreviations**
ALP: alkaline phosphatase
ALT: alanine aminotransferase
AMC: adverse muscle composition
ASAT: abdominal subcutaneous adipose tissue
AST: aspartate aminotransferase
BMI: body mass index
CAP: controlled attenuation parameter
DPP‐4 inhibitor: dipeptidyl peptidase‐4 inhibitor
FFMV: fat‐free muscle volume
FIB‐4: fibrosis‐4
HbA1c: hemoglobin A1c
HCC: hepatocellular carcinoma
HDL: high‐density lipoprotein
HOMA‐IR: homeostatic model assessment for insulin resistance
hsCRP: high‐sensitivity C‐reactive protein
INR: international normalized ratio
LDL: low‐density lipoprotein
LV concentricity: left ventricular concentricity
LVEDVi: left ventricular end‐diastolic volume index
LVEF: left ventricular ejection fraction
LVMi: left ventricular mass index
LVSVi: left ventricular stroke volume index
MFI: muscle fat infiltration
MRI: magnetic resonance imaging
NFS: nonalcoholic fatty liver disease fibrosis score
NPV: negative predictive value
PDFF: proton‐density fat fraction
PEth: phosphatidylethanol
PPV: positive predictive value
SGLT2: sodium‐glucose cotransporter‐2
T2D: Type 2 diabetes
VAT: visceral adipose tissue
VCTE: vibration‐controlled transient elastography
ɣGT: gamma‐glutamyl transferase

## Introduction

Metabolic dysfunction‐associated steatotic liver disease (MASLD) is the most common chronic liver disease worldwide, with an estimated global prevalence of 38% among adults [1, 2]. MASLD is often seen as the hepatic manifestation of the metabolic syndrome and is strongly associated with obesity and Type 2 diabetes mellitus (T2DM) [3]. The prevalence of MASLD in individuals with T2DM is approximately 55%–73% [4, 5]. Obesity, insulin resistance, and overconsumption of alcohol are important modifiers of the progression of the disease; therefore, patients with MASLD and T2DM have an increased risk of advanced fibrosis and hepatocellular carcinoma (HCC) [6, 7, 8, 9, 10]. Biopsy‐verified studies in patients with MASLD and T2DM have reported a prevalence of advanced fibrosis ranging from 40% to 60% [11, 12, 13].

Therefore, guidelines from the European Association for the Study of Diabetes, the European Society of the Study of Obesity, and the European Society for the Study of Liver recommend screening for MASLD in individuals with obesity or metabolic syndrome and targeted screening for metabolic dysfunction‐associated steatohepatitis (MASH) and liver fibrosis in high‐risk groups, including those aged >50 years and those with T2DM or metabolic syndrome [14]. In parallel, the American Association for the Study of Liver Diseases proposed that all patients with T2DM be screened for advanced fibrosis using the fibrosis‐4 (FIB‐4) score, and if it is elevated, proceed with vibration‐controlled transient elastography (VCTE), magnetic resonance elastography, or the enhanced liver fibrosis test [15].

However, the prevalence of advanced liver disease in individuals with T2DM mainly stems from studies conducted in tertiary care centers [16], inferring a selection bias for patients who are more prone to severe metabolic dysfunction and advanced liver disease. Therefore, we conducted a primary care‐based study on patients with T2DM using VCTE and magnetic resonance imaging (MRI) to evaluate the prevalence and severity of MASLD and advanced fibrosis. Furthermore, we examined the differences in MRI biomarkers, such as body composition and cardiac remodeling, and their associations with advanced fibrosis and hepatic fat infiltration in patients with and without MASLD.

## Materials and methods

### Study design and subjects

Patients with T2DM in Östergötland County attending annual checkups at their primary healthcare center between 2019 and 2023 were eligible for inclusion in the study. The patients were asked to participate in the study by their diabetes nurse or treating physician. Due to slow enrolment during the coronavirus disease 2019 pandemic, patients registered at primary healthcare centers were also invited to participate in the study through invitational letters. The inclusion criteria were as follows: (1) diagnosis of T2DM, according to current guidelines, and (2) age 35–75 years. The exclusion criteria were as follows: (1) contraindication for MRI (pacemaker, ferrous metal implants/fragments, claustrophobia, extreme obesity, and/or pregnancy), (2) alcohol dependence, (3) previously diagnosed liver cirrhosis, or (4) previously diagnosed primary liver disease, except MASLD. If they agreed to participate, they were referred to one of two study centers: the Department of Gastroenterology and Hepatology in Linköping, Sweden, or the Department of Medicine in Norrköping, Sweden.

At the study centers, patients were provided with information on the study and an opportunity to ask questions. Subsequently, all patients provided written informed consent, as witnessed and dated by a member of the research team.

### Ethics approval

Written informed consent was obtained from all participants prior to their inclusion in the study. The EPSONIP study was conducted in accordance with the principles outlined in the Declaration of Helsinki, approved by the Regional Ethical Board of Östergötland (2018/176‐31 and 2018/494‐32), and registered as a clinical trial at clinicaltrials.gov with the identifier NCT03864510. The study protocol has been previously published [17].

### Data collection and biochemical analysis

Following informed consent, a research nurse recorded the medical and family history of each participant, including current and recent pharmacological treatments. Consumption of alcohol was assessed through an oral interview and the Alcohol Use Disorder Identification Test‐Consumption [18]. Blood pressure, waist and hip circumference, body weight, and height were measured. Blood samples were collected from all participants for the analysis of routine biochemical parameters, as well as biobanking of serum, plasma, and whole blood.

Blood was collected after an overnight fast (minimum of 6 h) for a complete blood examination, analysis of prothrombin, iron, transferrin, transferrin saturation, ferritin, hemoglobin A1c (HbA1c), aspartate aminotransferase (AST), alanine aminotransferase (ALT), alkaline phosphatase (ALP), gamma‐glutamyl transferase (ɣGT), bilirubin, thyroid stimulating hormone, anti‐transglutaminase antibodies, total cholesterol, low‐ and high‐density lipoprotein (HDL), glucose, serum insulin, ceruloplasmin, phosphatidylethanol (PEth), and plasma protein electrophoresis, including, among others, albumin, α_1_‐antitrypsin, and immunoglobulins. In addition, blood was collected for detection of hepatitis B surface antigen (HBsAg), hepatitis B virus (HBV) DNA, and anti‐hepatitis C virus (HCV), antinuclear, smooth muscle, and anti‐mitochondrial antibodies. In addition, blood was collected from all participants with an elevated ferritin level to identify C282Y and H63D mutations in the *HFE* gene. In participants with a low α_1_‐antitrypsin level, the *S* and *Z* mutations in the *SERPINA1* (*Pi*) gene were analyzed.

Height and weight were measured with the participants dressed in light indoor clothing, without shoes. Waist circumference was measured midway between the hip bones and the bottom of the ribs, at the level of the umbilicus. Hip circumference was measured at the widest point of the buttocks. Overweight was defined as body mass index (BMI) ≥25 kg/m^2^ and <30 kg/m^2^, obesity as BMI ≥ 30 kg/m^2^, hypertension as blood pressure ≥130/85 mmHg, measured after the participant was lying down for at least 5 min, or requiring treatment, and hypertriglyceridemia as fasting triglycerides ≥150 mg/dL. Metabolic syndrome was defined according to the National Cholesterol Education Program–Adult Treatment Panel III definition [19], as having three of the following four factors: (1) waist circumference ≥40 in in males or ≥35 in in females; (2) fasting triglycerides ≥150 mg/dL or treatment for this lipid abnormality; (3) reduced fasting HDL <40 mg/dL in males or <50 mg/dL in females or treatment for this lipid abnormality; (4) systolic blood pressure ≥130 or diastolic blood pressure ≥85 mmHg or prescribed antihypertensive drug treatment; and (5) fasting plasma glucose ≥100 mg/dL or previously diagnosed T2DM. Homeostatic model assessment for insulin resistance (HOMA‐IR) was calculated in all participants who had not received insulin treatment according to the formula, fasting insulin (µU/L) × fasting glucose (mg/dL)/405. Overconsumption of alcohol was defined as a reported consumption of ≥210 g/week for males or ≥140 g/week for females.

Fibrosis scores were calculated using biochemical and clinical data as follows:

$$\begin{array}{ccc} \mathrm{FIB}-4 & = & \mathrm{age}\left[\mathrm{years}\right]\times \mathrm{AST}\left[U/L\right]/\\ & & \left(\mathrm{Platelet}\mathrm{count}\left[10^{9}/L\right]\times \surd \mathrm{ALT}\left[U/L\right]\right) \end{array}$$

Nonalcoholic fatty liver disease [NAFLD] fibrosis score (NFS) = −1.675 + (0.037 × age [years]) + (0.094 × BMI [kg/m^2^]) + (1.13 × T2D [yes = 1, no = 0]) + (0.99 × AST/ALT ratio) − (0.013 × platelet count [×10^9^/L]) − (0.66 × albumin [g/dL]).

### Vibration‐controlled transient elastography

VCTE, including controlled attenuation parameter (CAP), was performed using Fibroscan (Echosens) by experienced certified research nurses. As per the recommendations, all participants were asked to be in a fasting state of 3 h or longer. Furthermore, to ensure reliability, measurements required at least 10 valid readings, a success rate of ≥80%, and an interquartile range ≤30% of the median. Examinations began with the M probe and were switched to the XL probe, as recommended by the automated software.

### Liver biopsy

In participants with an elevated Fibroscan (VCTE ≥ 8 kPa), liver biopsy was recommended as part of the clinical routine. All liver biopsies were performed percutaneously with ultrasonography guidance and a 1.6 mm Biopince needle and were reviewed by an experienced liver histopathologist. All biopsy specimens were graded using the MASH‐FLIP algorithm [20]. Fibrosis was staged as described by Kleiner et al. [21].

### Magnetic resonance spectroscopy–proton‐density fat fraction (^1^H‐MRS‐PDFF)

^1^H‐magnetic resonance spectroscopy (MRS) was performed at 1.5 T (Philips Achieva dStream, Philips Healthcare) using a multi‐echo STEAM sequence with TM = 12 ms (shortest), fixed to minimize differential *J*‐coupling effects; TR = 3.200 ms; NSA = 1; FA = 90°, in combination with a series of TE = 10 to 35 ms, number of TEs = 6, TE spacing = 5 ms [22]. An experienced radiographer placed a 30 × 30 × 30 mm^3^ voxel of interest, avoiding the major vasculature in the liver. The acquisition was repeated twice. Post‐processing of the MRS data was performed by quantifying the integrals of water and fat resonances in jMRUI [23], using the AMARES algorithm [24]. Corrections for differences in T2 were performed. Quantification of the proton‐density fat fraction (PDFF) was performed using MATLAB (MathWorks), using the mean of the two acquisitions.

### Definitions of MASLD and suspected advanced fibrosis

MASLD was defined as PDFF ≥5%. In the absence of MRI‐specific evaluation of hepatic infiltration, a CAP value of ≥248 dB/m was used as the cut‐off for steatosis Grade 1 [25]. Suspected advanced fibrosis was defined as the liver stiffness measurement (LSM) as measured by a VCTE of ≥10 kPa.

### Left ventricular morphology and systolic function

Short axis images were acquired using a multi‐slice cine balanced steady state‐free precession sequence (TR 2.9 ms, TE 1.4 ms, flip angle 60°, compressed sensing factor 3, acquired spatial resolution 2.0 × 2.0–2.6 × 8.0 mm^3^, reconstructed spatial resolution 0.9 × 0.9 × 8.0 mm^3^, acquired temporal resolution 34 ms reconstructed to 30 timeframes) and post‐processed in software Segment Research version 12067b (Medviso AB) [26] to quantify left ventricular (LV) morphology and global systolic function. The LV endocardial and epicardial borders were automatically traced in short‐axis image stacks throughout the cardiac cycle to acquire mass, end‐diastolic volume, stroke volume, and ejection fraction. The ratio of mass to end‐diastolic volume was also calculated as the LV concentricity. Mass, end‐diastolic volume, and stroke volume were normalized to the body surface area and calculated using Mosteller's formula [27].

### Body composition

A dual‐echo Dixon MRI protocol, which provides a water‐ and fat‐separated volumetric data set covering the neck to the knees, as well as a multiecho Dixon acquisition for PDFF assessment of the liver, was used to analyze body composition; thigh fat‐free muscle volume (FFMV) and muscle fat infiltration (MFI), and visceral adipose tissue (VAT) and abdominal subcutaneous adipose tissue (ASAT) volumes, and liver PDFF using AMRA Researcher (AMRA Medical AB) [28]. The methodology and MRI protocol used in this study have been previously described [17]. Briefly, the analysis consisted of the following steps (based on the MR images): (1) automatic image calibration, (2) automatic labeling and registration of fat and muscle regions to the acquired image volumes, (3) quality control of anatomical regions and MR data performed by trained personnel at AMRA Medical AB, and (4) quantification of fat and muscle volumes based on the calibrated images [28, 29, 30, 31, 32]. These body composition measurements have been reported to be comparable across commonly used MRI scanners and field strengths [33].

For each participant, the muscle assessment score (MAsS) was calculated, consisting of a sex‐ and BMI‐invariant FFMV *z*‐score and MFI adjusted for sex differences using the median MFI in a sex‐specific reference population. Myosteatosis was defined as a high MFI (>75th percentile in a sex‐specific reference population), and low muscle volume was defined as a low FFMV *z*‐score (<25th percentile in a reference population). Participants with myosteatosis and low muscle volume were considered to have adverse muscle composition (AMC; defined by a combined high MFI and low FFMV *z*‐score) [34, 35, 36].

### Statistical analysis

Data are expressed as means and standard deviations or as numbers and percentages, where applicable. Categorical variables were compared using Pearson's chi‐square test. Continuous variables were compared using the Mann–Whitney *U*‐test or the Kruskal–Wallis test for more than two groups. Missing data points were handled using pairwise exclusion, without imputation. Univariable logistic regression analysis was used to assess the association between the potential risk factors and the likelihood of suspected advanced fibrosis. Odds ratios (ORs) with 95% confidence intervals (CIs) were calculated to estimate the strength of associations. Multivariable analysis was performed on variables deemed significant predictors in the crude analysis. Two‐sided *p‐*values were reported for all statistical tests, and *p* < 0.05 was considered to be statistically significant. All statistical analyses were conducted using IBM SPSS Statistics for Macintosh, Version 29.0.2.0 (IBM Corp).

## Results

### Study cohort and clinical characteristics

**Fig. 1:**
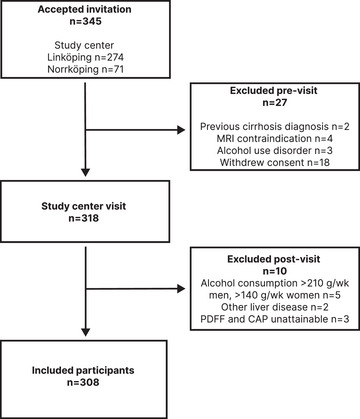
Flowchart of application of the inclusion and exclusion criteria. CAP, controlled attenuation parameter; MRI, magnetic resonance imaging; PDFF, proton‐density fat fraction.

Of the 345 individuals that accepted participation, 27 were excluded before the visit to the study center, and of these, 9 met ≥1 of the exclusion criteria and 18 withdrew their consent (Fig. 1).

After pre‐visit exclusion, 318 participants had a study center inclusion visit. Ten participants were excluded after the inclusion visit: five because of overconsumption of alcohol, three because the comorbid MASLD status could not be determined (they did not have ^1^H‐MRS‐PDFF, VCTE, or CAP), and two in whom another primary chronic liver disease was identified (one with α_1_ antitrypsin deficiency and one with pancreatic cancer with liver metastases).

In total, 308 participants were included in the final cohort, of whom 114 (37%) were female. Mean age was 63.9 ± 8.1 years, and the mean time since diagnosis of T2DM was 8.7 ± 6.8 years (range 0–35 years). Antidiabetic treatment/management was lifestyle modifications in 30 participants (10%), oral antidiabetic medication in 213 participants (69%), and insulin treatment in 65 participants (21%) (Table 1).

**Table 1:** Characteristics of the metabolic dysfunction‐associated steatotic liver disease (MASLD) and non‐MASLD groups (n = 308).

	All patients	Non‐MASLD *n* = 127	MASLD *n* = 181	
Parameter	[Mean ± SD or *n* (%)]	[Mean ± SD or *n* (%)]	[Mean ± SD or *n* (%)]	*p*
**Demographic and clinical**				
Sex (women)	114 (37%)	47 (37%)	67 (37%)	0.399
Age (years)	63.9 ± 8.1	64.3 ± 7.7	63.5 ± 8.3	0.451
Time since T2D diagnosis (years)	8.7 ± 6.8	8.8 ± 7.0	8.5 ± 6.7	0.839
BMI (kg/m^2^)	29.5 ± 4.6	27.4 ± 4.1	31.1 ± 4.4	**<0.001**
Overweight (25–30 kg/m^2^)	121 (39%)	60 (48%)	61 (34%)	**0.014**
Obesity (≥30 kg/m^2^)	135 (44%)	26 (21%)	109 (60%)	**<0.001**
Waist circumference (cm)	105.6 ± 12.6	99.8 ± 11.8	109.7 ± 11.6	**<0.001**
Waist to hip ratio	1.0 ± 0.1	0.97 ± 0.08	1.01 ± 0.07	**<0.001**
Alcohol (g/w)	28.7 ± 34.1	25.6 ± 29.0	31.0 ± 37.2	0.539
Hypertension	202 (66%)	77 (61%)	125 (69%)	0.125
Systolic BP (mm/Hg)	131.5 ± 15.1	130.8 ± 15.0	131.9 ± 15.2	0.378
Diastolic BP (mm/Hg)	78.5 ± 10.7	77.3 ± 9.8	79.5 ± 11.3	0.099
Manifest CVD	74 (24%)	29 (23%)	45 (25%)	0.991
Dyslipidemia	244 (79%)	103 (81%)	141 (78%)	0.495
Metabolic syndrome^a^	276 (92%)	112 (90%)	164 (94%)	0.370
Glucose lowering therapy				
Lifestyle modification	30 (10%)	13 (10%)	17 (9%)	0.941
Oral medication	213 (69%)	91 (72%)	122 (67%)	0.427
Insulin treatment	65 (21%)	23 (18%)	42 (23%)	0.281
Combination therapy	120 (39%)	44 (35%)	76 (42%)	0.193
Statins	226 (73%)	99 (78%)	127 (70%)	0.128
**Biochemical**				
INR	1.0 ± 0.2	1.0 ± 0.2	1.0 ± 0.1	**0.007**
Platelet count (10^9^/L)	230.0 ± 56.8	229.6 ± 54.5	230.3 ± 58.5	0.892
AST (U/L)	25.3 ± 7.9	23.6 ± 6.5	26.5 ± 8.5	**0.004**
ALT (U/L)	29.7 ± 15.7	23.9 ± 8.9	33.8 ± 18.0	**<0.001**
ɣGT (U/L)	37.1 ± 61.3	23.3 ± 22.8	46.8 ± 76.2	**<0.001**
Bilirubin (µmol/L)	9.3 ± 4.6	8.6 ± 4.0	9.8 ± 4.9	**0.021**
ALP (U/L)	69.6 ± 22.9	68.8 ± 18.3	70.3 ± 25.6	0.992
Albumin (g/L)	41.4 ± 4.5	41.6 ± 3.4	41.2 ± 5.1	0.850
fP‐Glucose (mg/dL)	140.0 ± 32.9	134.0 ± 29.1	144.2 ± 34.6	**0.001**
HbA1c (mmol/mol)	51.8 ± 11.6	49.8 ± 10.2	53.3 ± 12.4	**0.006**
Creatinine (µmol/L)	74.5 ± 19.6	74.1 ± 22.6	74.8 ± 17.3	0.376
Cholesterol (mmol/L)	4.0 ± 1.1	4.0 ± 1.1	4.0 ± 1.1	0.701
Triglycerides (mmol/L)	1.5 ± 1.3	1.1 ± 0.5	1.8 ± 1.6	**<0.001**
LDL (mmol/L)	2.0 ± 0.8	2.0 ± 0.8	2.0 ± 0.8	0.734
HDL (mmol/L)	1.3 ± 0.4	1.4 ± 0.4	1.3 ± 0.4	**<0.001**
hsCRP (mg/L)	2.5 ± 5.9	1.9 ± 3.6	2.9 ± 7.0	**<0.001**
f‐Insulin (mIE/L)^b^	16.0 ± 10.4	10.1 ± 5.3	20.4 ± 11.1	**<0.001**
f‐C‐peptide (nmol/L)	1.1 ± 0.5	0.8 ± 0.4	1.2 ± 0.5	**<0.001**
HOMA‐IR^c^	5.5 ± 4.2	3.2 ± 1.8	7.1 ± 4.6	**<0.001**
**Hepatic imaging**				
PDFF *n* = 282	8.3 ± 7.3	2.5 ± 1.2	12.9 ± 6.8	**<0.001**
VCTE *n = *304	6.0 ± 4.0	4.9 ± 1.4	6.8 ± 5.0	**<0.001**
≥8 kPa	43 (14%)	2 (1.6%)	41 (23%)	
≥10 kPa	20 (6.6%)		20 (11%)	
≥15 kPa	5 (1.6%)		5 (2.8%)	

*Note: p*‐values were calculated comparing MASLD and non‐MASLD groups. Categorical variables were tested with *χ* ^2^‐test and continual variables Mann–Whitney *U*‐test. Bold values indicate statistically significant differences.

Abbreviations: ALP, alkaline phosphatase; ALT, alanine aminotransferase; AST, aspartate aminotransferase; BMI, body mass index; CVD, cardiovascular disease; FIB‐4, fibrosis‐4; ɣGT, gamma‐glutamyl transferase; HbA1c, hemoglobin A1c; HDL, high‐density lipoprotein; HOMA‐IR, homeostatic model assessment for insulin resistance; hsCRP, high‐sensitivity C‐reactive protein; INR, international normalized ratio; LDL, low‐density lipoprotein; MASLD, metabolic dysfunction‐associated steatotic liver disease; PDFF, proton‐density fat fraction; T2D, Type 2 diabetes; VCTE, vibration‐controlled transient elastography.

^a^ *n* = 299.

^b^ *n* = 240.

^c^ *n* = 234.

### Prevalence and predictors of MASLD

Magnetic resonance‐based PDFF was available for 282 participants, 154 of whom had PDFF ≥5%. Of the 26 participants with missing MRI data, 5 had a contraindication for MRI, 16 did not undergo MRI due to claustrophobia, 3 were too large for the MRI machine, and for 2, investigation was not possible due to technical errors. Therefore, for the 26 participants without MRI, CAP was used, and 25 participants were deemed to have MASLD according to CAP (≥248 dB/m).

In the participants who underwent MRI but had a PDFF < 5%, two had a VCTE value ≥10 kPa, with no other primary liver disease, and were deemed to have MASLD.

**Fig. 2:**
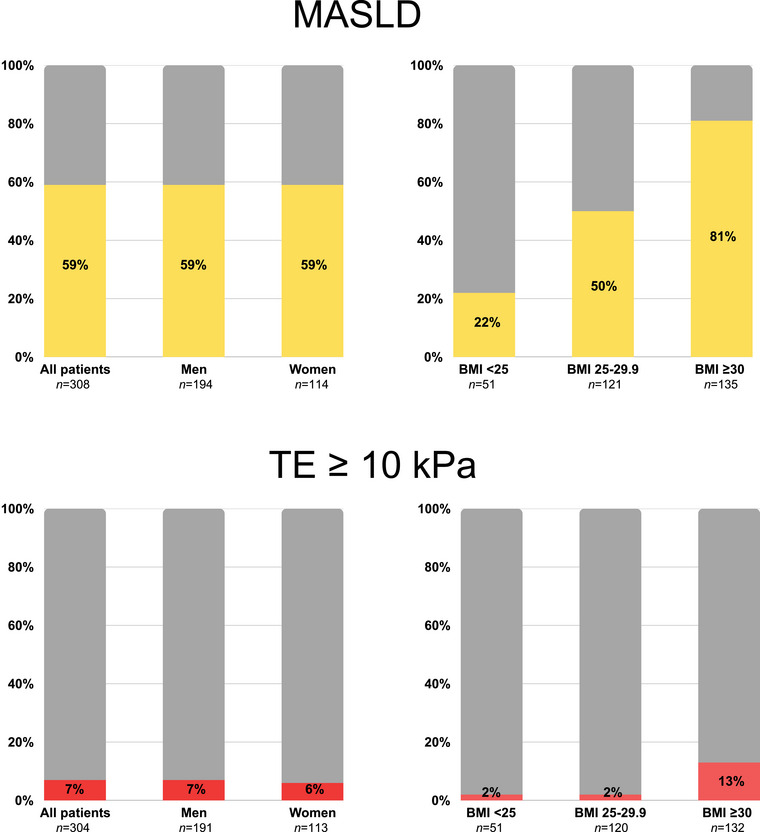
Prevalence of metabolic dysfunction‐associated steatotic liver disease (MASLD) and suspected advanced fibrosis (transient elastography [TE] ≥10 kPa) in all patients and by sex, and overweight or obesity status. BMI, body mass index.

Hence, in total, 181 (59%) participants were defined as having MASLD. The MASLD group had a greater mean BMI (31.1 ± 4.4 vs. 27.4 ± 4.1 kg/m^2^, *p* < 0.001) than the non‐MASLD group, with a higher frequency of obesity (60% vs. 21%, *p* < 0.001), and a higher waist circumference (109.7 ± 11.6 vs. 99.8 ± 11.8 cm, *p* < 0.001). In addition, the MASLD group had higher fasting insulin, C‐peptide, HbA1c, AST, ALT, and ɣGT levels than the non‐MASLD group (Table 1). There was no difference in sex, age, and antidiabetic or statin treatment, including glucagon‐like peptide analogues (Table S1), between the MASLD and non‐MASLD groups. The prevalence of MASLD across BMI categories and between sexes is illustrated in Fig. 2.

### Prevalence and predictors of suspected advanced fibrosis

VCTE was successful in 304 participants of whom 20 (7%) had suspected advanced fibrosis (LSM ≥ 10 kPa). Logistic regression analysis identified higher BMI, ASAT, ALT, AST, ɣGT, fasting insulin, HOMA‐IR, and C‐peptide as predictive of suspected advanced fibrosis. Each unit increase in BMI (kg/m^2^) was associated with 24% higher likelihood of suspected advanced fibrosis (crude OR [cOR] 1.24, 95% CI 1.13–1.37, *p* < 0.001). Additionally, obesity (BMI ≥ 30 kg/m^2^) was predictive, with participants with obesity having over eight times the likelihood of suspected advanced fibrosis compared to participants without obesity (cOR 8.28, 95% CI 2.37–28.90, *p* < 0.001) (Table S2).

In the multivariable analysis adjusted for age and sex, all identified predictors remained significant. However, after further adjustment for near‐significant variables (*p *< 0.10), excluding those with significant collinearity, only obesity (adjusted OR [aOR] 9.147, 95% CI 1.746–47.922, *p* < 0.01) and AST (U/L) (aOR 1.168, 95% CI 1.046–1.304, *p* < 0.01) were independent predictors of suspected advanced fibrosis.

In a subgroup analysis of participants with MASLD and available VCTE (*n *= 180), 11% had suspected advanced fibrosis (Table S3). The fibrotic MASLD group had higher BMI (34.2 ± 5.6 vs. 30.7 ± 4.0 kg/m^2^, *p* < 0.01), higher prevalence of obesity (85% vs. 57%, *p* < 0.05), and higher waist circumferences (117.4 ± 13.4 vs. 108.7 ± 11.1 cm, *p* < 0.01). Biochemically, the fibrotic MASLD group had higher AST (34.1 ± 13.1 vs. 25.5 ± 7.3 U/L, *p* < 0.01) and ɣGT (75.1 ± 137.9 vs. 43.5 ± 64.7 U/L, *p* < 0.05) levels.

The FIB‐4 score was higher in the fibrotic MASLD group compared to the non‐fibrotic MASLD group (1.67 ± 0.82 vs. 1.37 ± 0.51, *p* < 0.05), with no differences compared to the non‐MASLD group (1.47 ± 0.55) (Table S3). Using a higher FIB‐4 cut‐off (≥2.67), the positive predictive value (PPV) for suspected advanced fibrosis was 13%, whereas the negative predictive value (NPV) of FIB‐4 below the lower cut‐off (<1.30) was 96%.

Similarly, the NFS was higher in the fibrotic MASLD group (0.28 ± 1.12) compared with the non‐MASLD group (−0.31 ± 0.89, *p* < 0.05) and the non‐fibrotic MASLD group (−0.21 ± 0.94, *p* < 0.05). After applying the higher cut‐off (≥0.672), the PPV for suspected advanced fibrosis was 14%, whereas the NPV below the lower cut‐off (<−1.44) was 96%.

### Body composition and cardiac MRI

**Fig. 3:**
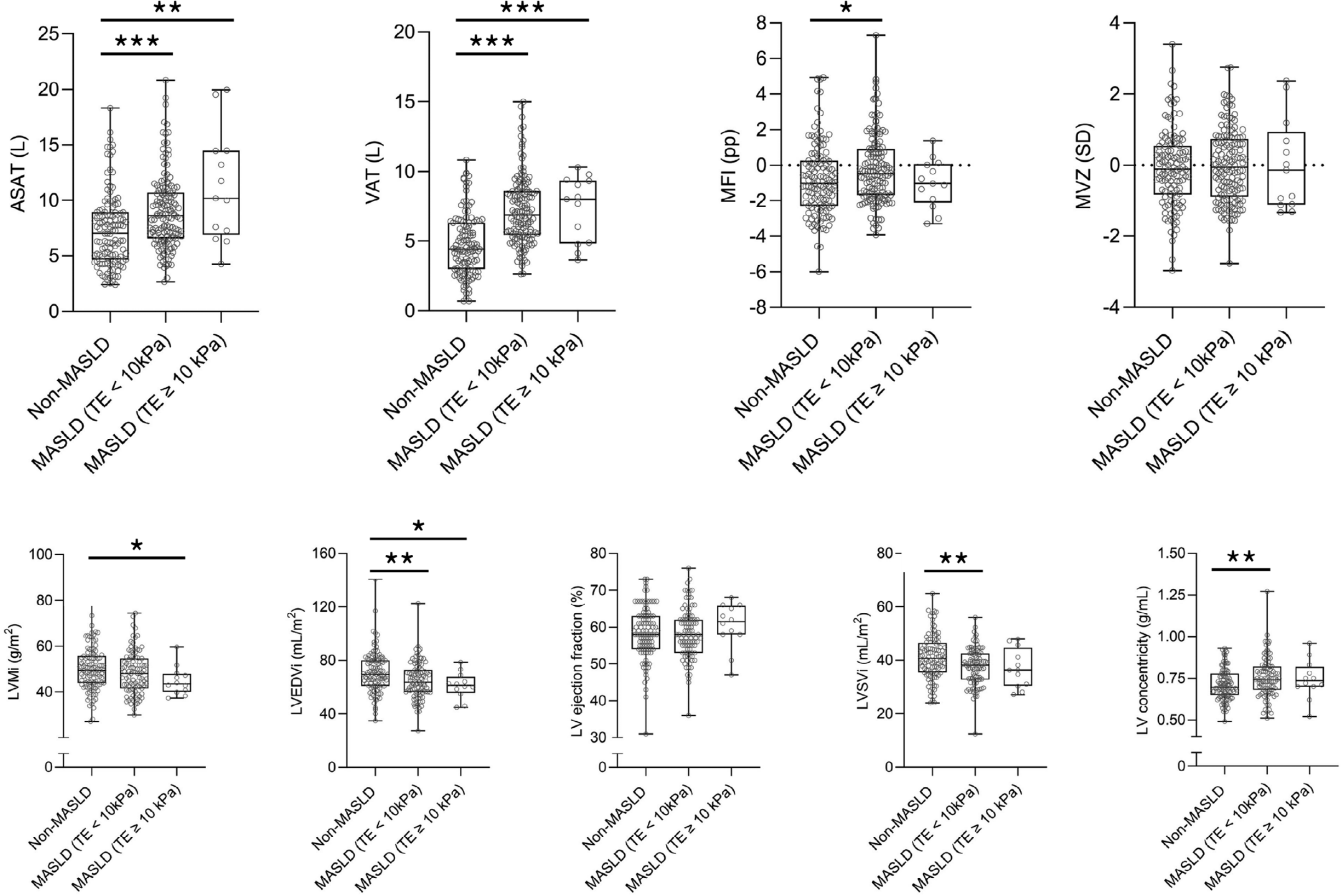
Abdominal subcutaneous adipose tissue (ASAT) and visceral adipose tissue (VAT) volume, muscle fat infiltration (MFI), muscle volume z‐score (MVZ), and cardiac magnetic resonance imaging in the metabolic dysfunction‐associated steatotic liver disease (MASLD) with suspected advanced fibrosis group (transient elastography [TE] ≥10 kPa), the MASLD group without fibrosis (TE < 10 kPa), and the non‐MASLD group. LV concentricity, left ventricular concentricity; LVEDVi, left ventricular end‐diastolic volume index; LVMi, left ventricular mass index; LVSVi, left ventricular stroke volume index. p‐values were calculated using the Mann–Whitney U‐test to compare the MASLD with suspected advanced fibrosis group, the MASLD without suspected advanced fibrosis group and the non‐MASLD group. ***p < 0.001. **p < 0.01. *p < 0.05.

In the body composition assessment, the non‐MASLD group had lower ASAT volumes than the non‐fibrotic and fibrotic MASLD groups. Similarly, VAT volume was lower in the non‐MASLD group than in the non‐fibrotic and fibrotic MASLD groups. Sex‐adjusted MFI was higher in the non‐fibrotic MASLD group compared to the non‐MASLD group (−0.21 ± 1.96 vs. −0.82 ± 2.04 pp, *p* < 0.05), whereas there were no other significant differences in MFI or FFMV *z*‐score observed when comparing the non‐MASLD, the non‐fibrotic MASLD, and the fibrotic MASLD groups. These data are illustrated in Fig. 3, and the exact variables are listed in Table S3.

For the presence of a high MFI, a low FFMV *z*‐score, and a combination, AMC was 35%, 31%, and 16%, respectively, in the entire cohort, with no significant differences between the non‐MASLD, the non‐fibrotic MASLD, and the fibrotic MASLD groups (Table S3).

In cardiac MRI, the LV stroke volume index was higher in the non‐MASLD group (41.2 ± 8.3 mL/m^2^) compared to the non‐fibrotic MASLD group (37.6 ± 7.0 mL/m^2^) (*p* < 0.01), and there were no significant differences when comparing either group to the fibrotic MASLD group (37.0 ± 7.3 mL/m^2^). The LV mass index was higher in the non‐MASLD group (50.0 ± 9.5 g/m^2^) compared to the fibrotic MASLD group (44.9 ± 6.3 g/m^2^, *p* < 0.05), with no differences when compared to the non‐fibrotic MASLD group (48.3 ± 9.3 g/m^2^). Meanwhile, LV concentricity was lower in the non‐MASLD group (0.71 ± 0.09 g/mL) compared to the non‐fibrotic MASLD group (0.76 ± 0.12 g/mL, *p* < 0.01), but there was no difference when compared to the fibrotic MASLD group (0.74 ± 0.12 g/mL) (Fig. 3).

LV end‐diastolic volume index was 71.2 ± 15.5 mL/m^2^ in the non‐MASLD group, 65.0 ± 13.8 mL/m^2^ in the non‐fibrotic MASLD group, and 61.2 ± 9.9 mL/m^2^ in the fibrotic MASLD group, with significant differences between the non‐MASLD group and the non‐fibrotic MASLD group (*p* < 0.01) and between the non‐MASLD group and the fibrotic MASLD group (*p* < 0.05).

LV ejection fraction was similar across groups, with values of 58.4% ± 7.0%, 58.4% ± 6.9%, and 60.5% ± 6.2%, in the non‐MASLD, the non‐fibrotic MASLD, and the fibrotic MASLD groups, respectively, with no significant differences.

### Histopathology

Of the 43 participants with VCTE ≥ 8 kPa, 21 underwent liver biopsy and 19 declined biopsy. Additionally, one participant had HCC and was assumed to have advanced fibrosis, and two had clinical signs of cirrhosis; liver biopsy was deemed contraindicated. Among participants who underwent biopsy, 4 (19%) had F0, 12 (57%) had non‐advanced fibrosis (F1–2), and 5 (24%) had advanced fibrosis (F3 *n = *4; F4 *n = *1) (Table S4). MASH was present in four participants who underwent liver biopsy, of whom one had fibrosis Stage 1, one had fibrosis Stage 2, and two had fibrosis Stage 3.

### Overweight, obesity, and prevalence of MASLD and suspected advanced fibrosis

Participants with obesity had a significantly higher prevalence of MASLD compared to those without obesity (81% vs. 42%, *p* < 0.001), and a markedly higher prevalence of fibrotic MASLD (13% vs. 2%, *p* < 0.001). Participants classified as overweight (i.e., BMI 25–30) had twice the prevalence of MASLD compared with those with a normal BMI (50% vs. 22%, *p* < 0.001). However, there was no significant difference in the prevalence of suspected advanced fibrosis between the overweight and normal BMI groups (2% vs. 2%) (Fig. 2).

### Phosphatidylethanol

Among the 300 participants with PEth and VCTE measurements available, 226 (75%) had a PEth value < 0.05 µmol/L (∼35 ng/mL), consistent with zero or undetectable alcohol consumption. Fourteen participants (5%) had a significantly elevated PEth level >0.30 µmol/L (∼211 ng/mL), indicating high consumption of alcohol. The remaining 60 (20%) had an intermediate PEth value between 0.05 and 0.30 µmol/L (∼35–211 ng/mL), suggesting moderate consumption of alcohol.

Among participants with suspected advanced fibrosis, five (25%) had an intermediate PEth level, whereas none had a significantly elevated level. Meanwhile, in participants without suspected advanced fibrosis, 55 (20%) had an intermediate PEth level, and 14 (5%) had a significantly elevated level. The prevalence of an elevated PEth level did not differ between participants with and without suspected advanced fibrosis.

## Discussion

### Main findings

In this prospective cohort study of 308 participants with T2DM from primary care, the prevalence of MASLD was 59%. The MASLD group had more cardiometabolic risk factors, including obesity and insulin resistance. Suspected advanced fibrosis was observed in 7% of all participants with T2DM and 11% of those with concomitant MASLD. Meanwhile, VCTE indicating cirrhosis (>15 kPa) was only observed in 1.6% (*n *= 5) of all participants with T2DM. Furthermore, clinical or histopathological signs of cirrhosis or HCC were determined in four participants (1.3%). Hence, in total, only 1.9% of all participants with T2DM had elastographic, histopathological, or radiological signs of cirrhosis.

In a previous study by Kwok et al., the prevalences of MASLD and suspected advanced fibrosis (>9.6 kPa) were 73% and 17.7%, respectively, in 1884 individuals with T2DM and reliable elastography [4]. The discordance in the study by Kwok et al., compared to our findings, could be a result of different sampling strategies, as in Kwok et al., the participants were recruited from primary and specialist care, but also because they used CAP for the diagnosis of MASLD. Similarly, a study by Michel et al. reported the prevalences of steatotic liver disease and suspected significant fibrosis as 77% and 42%, respectively. However, in that study, patients with MASLD, alcohol‐related liver disease, and metabolic and alcohol‐related liver disease were included [37].

The prevalences of MASLD and suspected advanced fibrosis reported in the present study are more in accordance with those reported by Forlano et al. and Ajmera et al. In the study by Forlano et al., the prevalence of ultrasonographic MASLD and suspected significant (>8.1 kPa) and suspected advanced (>12.1 kPa) fibrosis in 300 patients with T2DM, recruited from primary care, were 64%, 17%, and 11%, respectively [38]. Similarly, Ajmera et al. reported the prevalences of MRI‐defined MASLD and suspected advanced fibrosis (>9.7 kPa) as 65% and 14%, respectively, in 501 patients with T2DM, recruited from primary care and endocrinology clinics [39].

In the present study, obesity was a key risk factor for suspected advanced fibrosis, wherein the prevalence was 13% in participants with BMI ≥ 30 and only 2% in normal‐weight and overweight participants. Obese participants had an eightfold increase in the rate of suspected advanced fibrosis. Notably, in the adjusted analysis, obesity and the AST level were the only independent predictors of suspected advanced fibrosis. These findings align with those of previous studies on patients with T2DM, which also demonstrated a significantly higher risk of suspected advanced fibrosis in obese individuals than in those who were not obese [37, 39].

In our analysis, the FIB‐4 score and the NFS were suboptimal at ruling in and ruling out suspected advanced fibrosis. Using the higher cutoffs to rule in fibrosis, PPVs of 13% and 14% for the FIB‐4 score and NFS, respectively, were observed in this cohort with T2DM. Notably, the NPVs, when applying the lower cutoffs, were 96% for both scores, which offers limited value for ruling out participants unlikely to have advanced fibrosis, given that the prevalence of suspected advanced fibrosis in this cohort was 7%.

Another important finding was increased accumulation of ectopic fat in the MASLD group, including increased visceral and ASAT volume and MFI, whereas FFMV did not differ. Furthermore, the presence of AMC (low muscle volume and high MFI) was observed in 16% of the total cohort, with similar prevalences in the MASLD and non‐MASLD groups. At the same time, the fibrotic MASLD group had a twofold increased prevalence of AMC (31%), although this increase was not statistically significant. The reported prevalence of AMC in this study is somewhat higher than the 11% reported by Linge et al., in a study of 40,000 UK Biobank participants, but in concordance with the prevalence of AMC among 1200 participants with MASLD from the same cohort (14%) [34, 40].

Cardiac imaging revealed distinct LV characteristics in the MASLD group who had lower end‐diastolic volume, higher concentricity, and lower stroke volume but similar ejection fraction. These findings are in accordance with those of previous cardiac imaging studies on patients with MASLD, where higher liver fat content was linked to a small and concentric LV phenotype and, in echocardiographic studies, there was often diastolic impairment [41, 42, 43]. Evidence from a previous longitudinal study also supports MASLD as an independent risk factor for the development of heart failure, particularly heart failure with a preserved ejection fraction [44]. Although there was no difference in the prevalence of manifest cardiovascular disease between the MASLD and non‐MASLD groups, alterations in the LV characteristics in the MASLD group may reflect subclinical disease.

Participants with a previous diagnosis of alcohol use disorder or self‐reported overconsumption of alcohol were excluded. However, as no established PEth cutoff for MASLD has been identified, we did not exclude participants with an elevated PEth level from our analysis. Although we found the PEth level to be elevated in 5% of participants, the prevalence among those with suspected advanced fibrosis was not increased.

The main strength of our study is the comprehensive investigation of patients with T2DM, encompassing hepatic investigation of the prevalence of MASLD and severity and extra‐hepatic assessment, including neck‐to‐knee MRI‐based examination of muscle and body composition, including cardiac MRI, providing granular data. Furthermore, participants with mostly uncomplicated T2DM were included in this prospective population‐based primary care study. Moreover, for the diagnosis of MASLD, a reference method for quantitation of steatosis, ^1^H‐MRS‐PDFF, was used for the majority of the participants (92%) and the most validated method for elastographic measurements, Fibroscan.

This study has some limitations. First, the cohort may be subject to healthy volunteer bias, potentially underestimating severity of disease. Regarding generalizability, we acknowledge that the sample size was small; nevertheless, recruitment from multiple primary care centers in two different cities reduced the sampling bias in a non‐randomized setting. Furthermore, although all participants without obvious signs of cirrhosis, but with a transient elastography value ≥8 kPa, were given the option to undergo liver biopsy, only a subset of the participants accepted the procedure. In addition, in participants with elevated transient elastography, no repeat measurements were performed. A recent study by Lindfors et al. found that when repeating transient elastography in patients with an elevated LSM (≥8 kPa), 45% had normalized liver stiffness upon reassessment [45]. Furthermore, obesity as a predictor of a high LSM value could be a false‐positive secondary to the impact of obesity on LSM. Thus, the LSM value should be interpreted with caution in patients with obesity, which is also reflected in the Baveno VII guidelines [46]. Regarding the observation of alterations in the left ventricle in the MASLD group, but with no increase in the prevalence of manifest cardiovascular disease, it should be noted that the classification of manifest cardiovascular disease relied solely on medical history, which may have led to the underestimation of undiagnosed chronic heart failure or other cardiovascular conditions.

## Conclusions

In this cohort of highly phenotyped primary care patients with T2DM, 59% had MASLD, 14% had suspected significant fibrosis, and only 7% had suspected advanced fibrosis, as defined by elastography, and the predictors were obesity and AST. Absence of obesity is associated with a particularly low prevalence of elevated elastography. Compensated advanced chronic liver disease or cirrhosis was confirmed in only 1.9% of participants. The findings in this study suggest that advanced liver disease is rare in patients with T2DM who are receiving primary care, particularly the nonobese. In patients with obesity, >10% had suspected advanced fibrosis, suggesting that screening for fibrosis should focus on these patients. In addition, MASLD was associated with increased deposition of ectopic fat, including myosteatosis, but not with decreased muscle volume. We also found distinct alterations in the left ventricle in the MASLD group, which may reflect a higher prevalence of subclinical cardiovascular disease.

## Author contributions

*Study conception and design*: Mattias Ekstedt, Carl‐Johan Carlhäll, Tino Ebbers, Mikael Forsgren, Olof Dahlqvist Leinhard, Peter Lundberg, Stergios Kechagias, Patrik Nasr, and Nils Dahlström. *Acquisition of data*: All authors*. Statistical analysis*: Wile Balkhed, Patrik Nasr, and Mattias Ekstedt. *Analysis and interpretation of data*: Wile Balkhed, Patrik Nasr, and Mattias Ekstedt. *Drafting of manuscript*: Wile Balkhed, Patrik Nasr, and Mattias Ekstedt. *Critical revision*: All authors. All authors approved the final version of the article, including the authorship list.

## Conflict of interest statement

MF and ODL are employees and shareholders of AMRA Medical AB. ME has received lecture fees from Novo Nordisk. PL is a minority owner of AMRA Medical AB.

## Supporting information




**Supplementary Table 1**. Glucose lowering therapy in MASLD and non‐MASLD participants (*n* = 308).
**Supplementary Table 2**. Univariable and multivariable logistic regression analysis of predictors of suspected advanced fibrosis (VCTE ≥ 10 kPa).
**Supplementary Table 3**. Characteristics of non‐MASLD, MASLD with no fibrosis and MASLD with suspected advanced fibrosis (*n* = 304).
**Supplementary Table 4**. Fibrosis stage and vibration‐controlled transient elastography in participants with liver biopsy, clinical cirrhosis or HCC (*n* = 24).

## Data Availability

The data that support the findings of this study are available on request from the corresponding author. The data are not publicly available due to privacy or ethical restrictions.
