# Supplementary material for: Evaluating the prevalence and severity of metabolic dysfunction‐associated steatotic liver disease in patients with type 2 diabetes mellitus in primary care
Source: J Intern Med. 2025 Jun 16;298(3):173–87. doi: 10.1111/joim.20103 (PMC12374761; doi:10.1111/joim.20103)
Supplement: Supplementary file 1 — Supplementary Table 1. Glucose lowering therapy in MASLD and non‐MASLD participants (n = 308). Supplementary Table 2. Univariable and multivariable logistic regression analysis of predictors of suspected advanced fibrosis (VCTE ≥ 10 kPa). Supplementary Table 3. Characteristics of non‐MASLD, MASLD with no fibrosis and MASLD with suspected advanced fibrosis (n = 304). Supplementary Table 4. Fibrosis stage and vibration‐controlled transient elastography in participants with liver biopsy, clinical cirrhosis or HCC (n = 24). [file JOIM-298-173-s001.docx]

**Evaluating the prevalence and severity of MASLD in patients with type 2 diabetes mellitus in primary care**

Wile Balkhed, Martin Bergram, Fredrik Iredahl, Markus Holmberg, Carl Edin, Carl-Johan Carlhäll, Tino Ebbers, Pontus Henriksson, Christian Simonsson, Karin Rådholm, Gunnar Cedersund, Mikael Forsgren, Olof Dahlqvist Leinhard, Cecilia Jönsson, Peter Lundberg, Stergios Kechagias, Nils Dahlström, Patrik Nasr, Mattias Ekstedt

**Table of contents**
Supplementary Table 1............................................................................................................2
Supplementary Table 2............................................................................................................3
Supplementary Table 3............................................................................................................5
Supplementary Table 4............................................................................................................6

|  | **Supplementary table 1.** Glucose lowering therapy in MASLD and non-MASLD participants (*n=*308) | | | | |
| --- | --- | --- | --- | --- | --- |
| Parameter | | All patients | Non-MASLD  *n=*127 | MASLD  *n=*181 | ***P**** |
|  | | *n* (%) | *n* (%) | *n* (%) |  |
| Metformin | | 235 (76%) | 93 (73%) | 142 (78%) | .289 |
| Insulin | | 65 (21%) | 23 (18%) | 42 (23%) | .281 |
| Sulfonylurea | | 27 (9%) | 12 (9%) | 15 (8%) | .723 |
| DPP-4 inhibitor | | 5 (2%) | 3 (2%) | 2 (1%) | .390 |
| GLP-1 agonist | | 19 (6%) | 5 (4%) | 14 (8%) | .173 |
| SGLT2 inhibitor | | 86 (28%) | 35 (28%) | 51 (28%) | .905 |
| Pioglitazone | | 1 (.3%) | 1 (1%) | 0 | .232 |
| Monotherapy | | 156 (51%) | 70 (55%) | 86 (48%) | .189 |
| Combination therapy | | 120 (39%) | 44 (35%) | 76 (42%) | .193 |
| DPP-4 inhibitor, dipeptidyl peptidase-4 inhibitor; GLP-1, glucagon-like peptide-1; SGLT2, sodium-glucose cotransporter-2  ***P***-values were calculated comparing MASLD and non-MASLD groups and tested with χ^2^-test | | | | | |

| **Supplementary table 2.** Univariable and multivariable logistic regression analysis of predictors of suspected advanced fibrosis (VCTE ≥ 10 kPa) | | | | | | | | | |
| --- | --- | --- | --- | --- | --- | --- | --- | --- | --- |
|  | Model 1* | | | Model 2† | | | Model 3‡ | | |
| Variables | cOR | 95% CI | ***P*** | OR | 95% CI | ***P*** | OR | 95% CI | ***P*** |
| Age | 0.956 | 0.911-1.004 | .074 |  |  |  |  |  |  |
| Sex (women) | 0.709 | 0.264-1.900 | .494 |  |  |  |  |  |  |
| **BMI** | **1.240** | **1.125-1.367** | **<.001** | **1.253** | **1.125-1.395** | **<.001** |  |  |  |
| **Obesity (BMI ≥ 30)** | **8.278** | **2.372-28.897** | **<.001** | **7.741** | **2.198-27.257** | **.001** | **9.147** | **1.746-47.922** | **.009** |
| **ALT (U/L)** | **1.039** | **1.018-1.060** | **<.001** | **1.036** | **1.013-1.060** | **.002** | 0.984 | 0.921-1.051 | .622 |
| **AST (U/L)** | **1.112** | **1.061-1.165** | **<.001** | **1.107** | **1.054-1.162** | **<.001** | **1.168** | **1.046-1.304** | **.006** |
| HbA1c (mmol/mol) | 1.006 | 0.969-1.043 | .767 |  |  |  |  |  |  |
| **ɣGT (U/L)** | **1.005** | **1.001-1.009** | **.021** | **1.004** | **1.000-1.008** | **.048** | 0.998 | 0.993-1.004 | .604 |
| **f-Insulin (mIE/L)** | **1.056** | **1.018-1.095** | **.003** | **1.051** | **1.010-1.093** | **.014** |  |  |  |
| **f-C-peptide (nmol/L)** | **2.461** | **1.154-5.249** | **.020** | **2.421** | **1.146-5.115** | **.021** |  |  |  |
| **HOMA-IR** | **1.143** | **1.048-1.237** | **.003** | **1.128** | **1.028-1.237** | **.011** | 1.010 | 0.859-1.188 | .904 |
| **ASAT (L)** | **1.204** | **1.056-1.373** | **.006** | **1.256** | **1.052-1.500** | **.012** |  |  |  |
| VAT (L) | 1.180 | 0.971-1.433 | .097 |  |  |  |  |  |  |
| Sex-adjusted MFI (pp) | 0.867 | 0.639-1.175 | .358 |  |  |  |  |  |  |
| FFMV Z-score (SD) | 1.047 | 0.622-1.763 | .863 |  |  |  |  |  |  |
| High MFI | 0.860 | 0.258-2.867 | .806 |  |  |  |  |  |  |
| Low FFMV Z-score | 2.044 | 0.666-6.277 | .212 |  |  |  |  |  |  |
| AMC | 2.564 | 0.753-8.736 | .132 |  |  |  |  |  |  |
| LVMi (g/m^2^) | 0.945 | 0.880-1.014 | .117 |  |  |  |  |  |  |
| LVEDVi (mL/m^2^) | 0.963 | 0.920-1.008 | .103 |  |  |  |  |  |  |
| LVEF (%) | 1.048 | 0.959-1.145 | .303 |  |  |  |  |  |  |
| LVSVi (mL/m^2^) | 0.960 | 0.890-1.037 | .301 |  |  |  |  |  |  |
| LV concentricity (g/mL) | 2.617 | 0.015-466.246 | .716 |  |  |  |  |  |  |
| Abbreviations: ALT, alanine aminotransferase; AMC, adverse muscle composition; ASAT, abdominal subcutaneous adipose tissue; AST, aspartate aminotransferase; BMI, body mass index; FFMV, fat-free muscle volume; HbA1c, hemoglobin A1c; HOMA-IR, homeostatic model assessment for insulin resistance; LV concentricity, left ventricular concentricity; LVEDVi, left ventricular end-diastolic volume index; LVEF, left ventricular ejection fraction; LVMi, left ventricular mass index; LVSVi, left ventricular stroke volume index; MFI, muscle fat infiltration; VAT, visceral adipose tissue; VCTE, vibration-controlled transient elastography; ɣGT, gamma-glutamyl transferase  * Univariable unadjusted analysis  † Multivariable analysis adjusted for age and sex  ‡ Multivariable analysis adjusted for age, sex and all variables with p < 0.10, excluding those with significant collinearity | | | | | | | | | |

|  | **Supplementary table 3.** Characteristics of non-MASLD, MASLD with no fibrosis and MASLD with suspected advanced fibrosis (*n=*304) | | | | | | | |
| --- | --- | --- | --- | --- | --- | --- | --- | --- |
| Parameter | | Non-MASLD  *n=*124 | MASLD (VCTE  < 10kPa) *n=*160 | VCTE ≥ 10 kPa  *n=*20 |  |  |  |  |
| **Demographic and clinical** | | [Mean ± SD or *n* (%)] | [Mean ± SD or *n* (%)] | [Mean ± SD or *n* (%)] | ***P**** | 1 *vs.* 2 | 1 *vs.* 3 | 2 *vs.* 3 |
| Sex (women) | | 46 (37%) | 61 (38%) | 6 (30%) | .778 |  |  |  |
| Age (years) | | 64.4 ± 7.6 | 63.9 ± 7.7 | 60.8 ± 11.9 | .491 |  |  |  |
| Time since diagnosis (years) | | 8.7 ± 7.0 | 8.7 ± 6.6 | 7.3 ± 7.3 | .529 |  |  |  |
| BMI (kg/m^2^) | | 27.3 ± 4.0 | 30.7 ± 4.0 | 34.2 ± 5.6 | **<.001** | **<.001** | **<.001** | **.004** |
| Overweight (25-30 kg/m^2^) | | 59 (48%) | 59 (37%) | 2 (10%) | **.003** | .061 | **.001** | **.017** |
| Obesity (≥ 30 kg/m^2^) | | 24 (19%) | 91 (57%) | 17 (85%) | **<.001** | **<.001** | **<.001** | **.015** |
| Waist circumference (cm) | | 99.4 ± 11.4 | 108.7 ± 11.1 | 117.4 ± 13.4 | **<.001** | **<.001** | **<.001** | **.002** |
| Waist to hip ratio | | 0.97 ± 0.08 | 1.01 ± 0.07 | 1.03 ± 0.08 | **<.001** | **<.001** | **.018** | .439 |
| Alcohol (g/w) | | 25.8 ± 29.2 | 30.8 ± 35.9 | 34.1 ± 49.3 | .814 |  |  |  |
| Hypertension | | 76 (61%) | 111 (69%) | 13 (65%) | .362 |  |  |  |
| Systolic BP (mm Hg) | | 130.7 ± 15.2 | 131.9 ± 15.3 | 133.3 ± 13.9 | .552 |  |  |  |
| Diastolic BP (mm Hg) | | 77.3 ± 9.9 | 79.0 ± 11.0 | 83.6 ± 12.5 | .095 | .195 | **.039** | .171 |
| Manifest CVD | | 27 (22%) | 34 (21%) | 6 (30%) | .670 |  |  |  |
| Dyslipidemia | | 100 (81%) | 125 (78%) | 15 (75%) | .791 |  |  |  |
| Metabolic syndrome† | | 109 (90%) | 144 (94%) | 19 (95%) | .563 |  |  |  |
| Glucose lowering therapy | |  |  |  |  |  |  |  |
| Lifestyle modification | | 13 (10%) | 17 (11%) | 2 (10%) | .996 |  |  |  |
| Oral medication | | 89 (72%) | 109 (68%) | 13 (65%) | .728 |  |  |  |
| Insulin treatment | | 22 (18%) | 36 (23%) | 5 (25%) | .549 |  |  |  |
| Combination therapy | | 43 (35%) | 65 (41%) | 10 (50%) | .338 |  |  |  |
| Statin treatment | | 96 (77%) | 112 (70%) | 14 (70%) | .358 |  |  |  |
| FIB-4 | | 1.47 ± 0.55 | 1.37 ± 0.51 | 1.67 ± 0.82 | .072 | .146 | .147 | **.048** |
| FIB-4 < 1.30 | | 59 (48%) | 81 (51%) | 6 (30%) | .198 |  |  |  |
| FIB-4 1.30-2.67 | | 59 (48%) | 75 (47%) | 13 (65%) | .325 |  |  |  |
| FIB-4 ≥ 2.67 | | 5 (4%) | 2 (1%) | 1 (5%) | .280 |  |  |  |
| NAFLD Fibrosis Score | | -0.31 ± 0.89 | -0.21 ± 0.94 | 0.28 ± 1.12 | **.043** | .364 | **.012** | **.039** |
| NFS < -1.44 | | 9 (7%) | 15 (9%) | 1 (5%) | .699 |  |  |  |
| NFS -1.44-0.672 | | 97 (80%) | 117 (74%) | 12 (60%) | .148 |  |  |  |
| NFS ≥ 0.672 | | 16 (13%) | 26 (16%) | 7 (35%) | .**049** | .438 | **.014** | **.044** |
| **Biochemical** | |  |  |  |  |  |  |  |
| INR | | 1.0 ± 0.2 | 1.0 ± 0.1 | 1.0 ± 0.1 | **.010** | **.004** | .921 | .141 |
| Platelet count (10^9^/L) | | 230.9 ± 54.2 | 232.7 ± 58.5 | 212.4 ± 58.2 | .403 |  |  |  |
| AST (U/L) | | 23.6 ± 6.6 | 25.5 ± 7.3 | 34.1 ± 13.1 | **<.001** | **.029** | **<.001** | **.008** |
| ALT (U/L) | | 23.7 ± 8.7 | 32.5 ± 16.3 | 44.0 ± 26.9 | **<.001** | **<.001** | **<.001** | .079 |
| ɣGT (U/L) | | 23.3 ± 23.1 | 43.5 ± 64.7 | 75.1 ± 137.9 | **<.001** | **<.001** | **<.001** | **.017** |
| Bilirubin ( µmol/L) | | 8.7 ± 4.0 | 9.6 ± 4.8 | 11.2 ± 6.0 | **.048** | **.032** | .089 | .415 |
| ALP (U/L) | | 68.6 ± 18.4 | 69.9 ± 26.5 | 74.5 ± 16.2 | .205 |  |  |  |
| Albumin (g/L) | | 41.7 ± 3.3 | 41.4 ± 4.4 | 39.8 ± 9.2 | .888 |  |  |  |
| fP-Glucose (mg/dL) | | 133.9 ± 29.3 | 144.6 ± 35.4 | 144.1 ± 27.1 | **.003** | **.001** | .061 | .837 |
| HbA1c (mmol/moL) | | 49.7 ± 10.3 | 53.3 ± 12.5 | 52.6 ± 12.3 | **.020** | **.005** | .293 | .811 |
| Creatinine (µmol/L) | | 74.6 ± 22.5 | 74.7 ± 16.8 | 75.1 ± 22.1 | .599 |  |  |  |
| Cholesterol (mmol/L) | | 4.0 ± 1.2 | 4.0 ± 1.0 | 4.0 ± 1.0 | .776 |  |  |  |
| f-Triglycerides (mmol/l) | | 1.1 ± 0.5 | 1.8 ± 1.7 | 1.6 ± 0.7 | **<.001** | **<.001** | **<.001** | .741 |
| LDL (mmol/L) | | 2.0 ± 0.9 | 1.9 ± 0.8 | 2.2 ± 0.9 | .585 |  |  |  |
| HDL (mmol/L) | | 1.4 ± 0.4 | 1.3 ± 0.4 | 1.1 ± 0.3 | **<.001** | **<.001** | **<.001** | .103 |
| hsCRP (mg/L) | | 1.9 ± 3.6 | 2.7 ± 7.0 | 4.4 ± 7.8 | **<.001** | **<.001** | **<.001** | **.037** |
| f-Insulin (mIE/L)‡ | | 10.1 ± 5.4 | 19.8 ± 9.9 | 24.7 ± 18.5 | **<.001** | **<.001** | **<.001** | .487 |
| f-C-peptide (nmol/) | | 0.8 ± 0.4 | 1.2 ± 0.5 | 1.3 ± 0.5 | **<.001** | **<.001** | **<.001** | .402 |
| HOMA-IR§ | | 3.3 ± 1.8 | 6.9 ± 4.2 | 9.1 ± 7.2 | **<.001** | **<.001** | **<.001** | .464 |
| **Imaging** | |  |  |  |  |  |  |  |
| **Hepatic (*n=*278)** | |  |  |  |  |  |  |  |
| PDFF (%) | | 2.5 ± 1.2 | 12.9 ± 6.7 | 13.2 ± 8.3 | **<.001** | **<.001** | **<.001** | .925 |
| **Muscle and adipose tissue composition (*n=*280)** | |  |  |  |  |  |  |  |
| ASAT (L) | | 7.32 ± 3.33 | 9.00 ± 3.41 | 11.20 ± 4.98 | **<.001** | **<.001** | **.005** | .122 |
| VAT (L) | | 4.72 ± 2.22 | 7.20 ± 2.44 | 7.32 ± 2.34 | **<.001** | **<.001** | **<.001** | .670 |
| Sex-adjusted MFI (pp) | | -0.82 ± 2.04 | -0.21 ± 1.96 | -1.01 ± 1.37 | **.036** | **.014** | .944 | .210 |
| FFMV Z-score (SD) | | -0.09 ± 1.09 | -0.02 ± 1.02 | 0.00 ± 1.30 | .858 |  |  |  |
| High MFI | | 37 (30%) | 54 (38%) | 4 (31%) | .430 |  |  |  |
| Low FFMV Z-score | | 35 (29%) | 43 (30%) | 6 (46%) | .442 |  |  |  |
| AMC | | 14 (12%) | 25 (17%) | 4 (31%) | .133 |  |  |  |
| **Cardiac MRI (*n=*211)** | |  |  |  |  |  |  |  |
| LVMi (g/m^2^) | | 50.0 ± 9.5 | 48.3 ± 9.3 | 44.9 ± 6.3 | .079 | .162 | **.036** | .203 |
| LVEDVi (mL/m^2^) | | 71.2 ± 15.5 | 65.0 ± 13.8 | 61.2 ± 9.9 | **.002** | **.003** | **.014** | .385 |
| LVEF (%) | | 58.4 ± 7.0 | 58.4 ± 6.9 | 60.5 ± 6.2 | .424 |  |  |  |
| LVSVi (mL/m^2^) | | 41.2 ± 8.3 | 37.6 ± 7.0 | 37.0 ± 7.3 | **.008** | **.004** | .107 | .678 |
| LV concentricity (g/mL) | | 0.71 ± 0.09 | 0.76 ± 0.12 | 0.74 ± 0.12 | **.011** | **.003** | .214 | .882 |
| Abbreviations: ALP, alkaline phosphatase; ALT, alanine aminotransferase; AMC, adverse muscle composition; ASAT, abdominal subcutaneous adipose tissue; AST, aspartate aminotransferase; BMI, body mass index; CAP, controlled attenuation parameter; CVD, cardiovascular disease; FFMV, fat-free muscle volume; FIB-4, fibrosis-4; HbA1c, hemoglobin A1c; HDL, high-density lipoprotein; HOMA-IR, homeostatic model assessment for insulin resistance; hsCRP, high-sensitivity C-reactive protein; INR, international normalized ratio; LDL, low-density lipoprotein; LVEF, left ventricular ejection fraction; LV concentricity, left ventricular concentricity; LVEDVi, left ventricular end-diastolic volume index; LVMi, left ventricular mass index; LVSVi, left ventricular stroke volume index; MFI, muscle fat infiltration; NFS; NAFLD Fibrosis Score; PDFF, proton density fat fraction; VAT, visceral adipose tissue; VCTE, vibration-controlled transient elastography; ɣGT, gamma-glutamyl transferase  ****P***-values were calculated for comparisons among non-MASLD (Group 1), MASLD no fibrosis (Group 2), and MASLD with fibrosis (Group 3). Categorical variables were tested with χ^2^-test, with pairwise comparisons performed when significant differences (p < 0.10 were found). Continuous variables were assessed with Kruskal-Wallis test, with pairwise comparisons performed using Mann-Whitney U test when significant differences (p < 0.10) were found. Bold values indicate statistically significant differences. | | | | | | | | |

† *n*=295

‡ *n=*238

§ *n=*233

| **Supplementary table 4.** Fibrosis stage and vibration-controlled transient elastography in participants with liver biopsy, clinical cirrhosis or HCC (*n* = 24) | |
| --- | --- |
| **Fibrosis stage (Kleiner)** | **VCTE avg (spread)** |
| **F0 *n* = 4** | 10.3 (8.4 – 13.7) |
| **F1 *n* = 5** | 9.8 (8.8 – 11.4) |
| **F2 *n* = 7** | 9.6 (6.1 – 13.7) |
| **F3 *n* = 4** | 15.7 (9.7 – 30.1) |
| **F4 *n* = 1** | 16.3 |
| **Clinical cirrhosis *n* = 2** | 32.6 (10.6 – 54.6) |
| **HCC *n* = 1** | 13.0 |
